# Noble Metal-Based Nanocomposites for Surface-Enhanced Raman Spectroscopy Detection of Food Contaminants

**DOI:** 10.3390/foods14173108

**Published:** 2025-09-05

**Authors:** Huilin Li, Rui Gao, Xiaochun Hu, Mengmeng Gao, Mingfei Pan

**Affiliations:** 1 Key Laboratory of Food Quality and Health of Tianjin, Tianjin University of Science and Technology, Tianjin 300457, China; lhltust@163.com (H.L.); gaorui20011024@163.com (R.G.); l17753795263@163.com (X.H.); lhk2131@163.com (M.G.); 2 State Key Laboratory of Food Nutrition and Safety, Tianjin University of Science & Technology, Tianjin 300457, China

**Keywords:** noble metal-based nanocomposites, SERS detection, food contaminants

## Abstract

Public health concerns related to food contaminants, including biotoxins, pesticide and veterinary drug residues, illegal additives, foodborne pathogens, and heavy metals, have garnered significant public attention in recent years. Consequently, there is an urgent need to develop rapid and accurate technologies to detect these harmful substances. Surface-enhanced Raman spectroscopy (SERS), due to its characteristics of high sensitivity and specificity enabling the detection of food contaminants within complex matrices, has attracted widespread interest. This review focuses on the application of noble metal-based nanocomposites as SERS-active substrates for food contaminant detection. It particularly highlights the structure–performance relationships of metallic nanomaterials, including gold and silver nanoparticles (e.g., nanospheres, nanostars, nanorods), bimetallic structures (e.g., Au@Ag core–shell), as well as metal–nonmetal composite nanomaterials such as semiconductor-based, carbon-based, and porous framework-based materials. All of which play a crucial role in achieving effective Raman signal enhancement. Furthermore, the significant applications in detecting various contaminants and distinct advantages in terms of the sensitivity and selectivity of noble metal-based nanomaterials are also discussed. Finally, this review addresses current challenges associated with SERS technology based on noble metal-based nanomaterials and proposes corresponding strategies alongside future perspectives.

## 1. Introduction

In recent years, food safety issues have garnered global attention due to their increasing complexity and serious public health impacts. The World Health Organization (WHO) reports that nearly 600 million people worldwide fall ill annually from consuming food contaminated with chemical and biological pollutants, resulting in about 420,000 deaths [1]. This dramatic rise in foodborne diseases poses significant threats to both public health and economic stability. Food contaminants are broadly categorized as chemical and biological. Chemical contaminants, including biotoxins, antibiotics, pesticides/veterinary drugs, heavy metals and nitrites, can accumulate in tissues over time, leading to chronic toxicity. Their adverse effects include organ damage, neurotoxicity, and carry risks of teratogenicity, carcinogenicity, and mutagenicity. Biological contaminants primarily consist of pathogenic bacteria and viruses, such as *Salmonella*, *Escherichia coli*, and norovirus, which are associated with acute infections, toxin-mediated diseases, and occasional outbreaks [2,3]. Given the unpredictable and diverse nature of these contaminants, it is imperative to develop efficient and accurate sensing techniques for detecting food contaminants.

Currently, conventional methods applied for food contaminant analysis mainly include liquid chromatography (LC) [4], mass spectrometry (MS) [5], high-performance liquid chromatography-mass spectrometry (HPLC-MS) [6], polymerase chain reaction (PCR) [7], and biochemical assays. These methods are considered the gold standard for laboratory quantification due to their high accuracy. However, they often require cumbersome and time-costly pretreatment process, and rely on expensive instrumentation and specialized technical personnel. Consequently, developing novel food contaminant detection technologies characterized by high accuracy, sensitivity, and rapidity is significantly crucial. In contrast, SERS offers unique advantages for rapid screening, including fingerprint identification capability for highly sensitive and specific detection, as well as potential for rapid, on-site, and non-destructive analysis. Raman spectroscopy utilizes inelastic light scattering to identify molecular structures and characterize compositions through spectral fingerprints. However, its inherent weak signal restricts applications to solid or high-concentration solutions and impedes reliable quantification [8,9]. In 1974, Fleischmann et al. observed a dramatically enhanced Raman signal from pyridine adsorbed on a roughened silver (Ag) electrode [10]. This signal amplification phenomenon was subsequently termed surface-enhanced Raman spectroscopy (SERS) [11]. The intrinsic plasmonic properties of gold (Au) or Ag nanostructures render them common and efficient SERS substrates. Nevertheless, the dynamic environment of fluid samples restricts consistent contact between target molecules and plasmonic nanostructures, leading to poor reproducibility in SERS measurements [12,13]. Therefore, incorporating non-noble metal nanomaterials, which offer selective capture and rapid enrichment capabilities, can significantly improve SERS performance compared to monometallic noble metal substrates.

Numerous reviews on SERS technology for food contaminant analysis have recently emerged. These primarily discuss aptamer-based [14], antibody-based [15,16], and metal–organic framework (MOF)-based [17] SERS sensing strategies. Aptamers provide benefits such as high affinity and design flexibility, yet their conformational stability is strongly influenced by environmental conditions like pH and ionic strength [18]. Antibodies exhibit strong specificity, yet their high production cost and susceptibility to denaturation limit their practicality for on-site rapid detection. Offer exceptional capabilities for target enrichment owing to their ultrahigh surface area and tunable pore sizes. However, their typically small apertures may hinder the diffusion and capture of larger molecules, including certain protein toxins or pathogens, thereby reducing detection sensitivity. Integrating noble metals with functional non-metallic components enables precise nano-environment engineering to control analyte–substrate interactions. By incorporating recognition elements, such as aptamers, antibodies, and molecularly imprinted polymers (MIPs), targets can be selectively captured. Additionally, porous frameworks or magnetic cores facilitate pre-concentration, significantly increasing local analyte concentrations at SERS-active sites. This directed interaction markedly reduces variability and enhances detection reliability [19,20].

**Figure 1:**
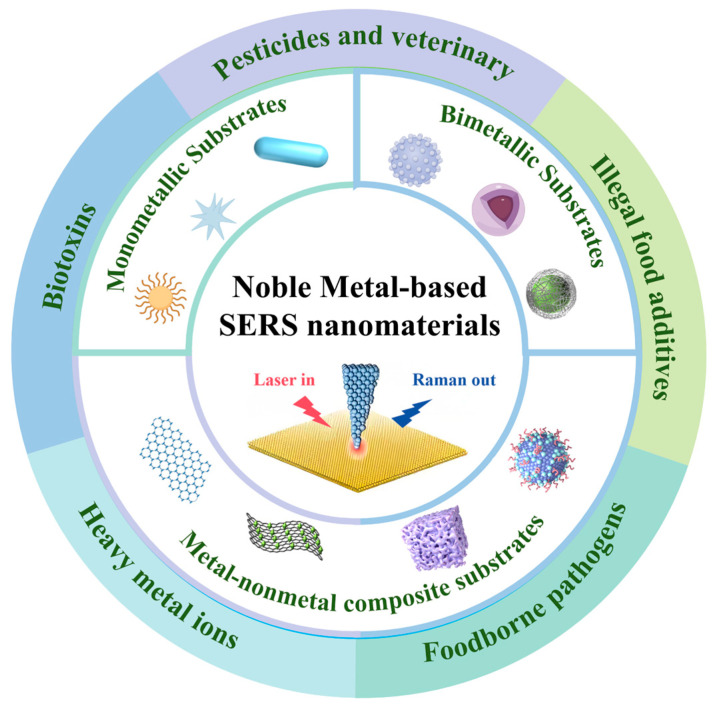
Classifications of noble metal-based SERS nanomaterials and their applications in food contaminants detection.

This review aims to fill that gap by comprehensively outlining noble metal nanocomposites as SERS substrates and their applications for detecting food contaminants (Figure 1). We critically examine categories of these nanocomposites and their role in improving the sensitivity and specificity of SERS technology. Challenges and future prospects of SERS detection strategies are further discussed. This paper offers effective solutions to multiple challenges in the practical application of SERS technology, including low signal reproducibility, strong matrix interference, and poor analytical robustness.

## 2. SERS-Active Substrate Materials

Traditional Raman spectroscopy is limited by a small scattering cross-section, producing extremely weak signals typically on the order of 10^−10^ of the incident light intensity. Moreover, SERS signals exhibit significant vulnerability to fluorescence-derived interference and laser-induced noise. To address these issues, SERS technology, based on electromagnetic enhancement (EM) and chemical enhancement (CM) mechanisms, has emerged [21]. EM arises primarily from plasmon-mediated field amplification at metallic nanostructures, where light-induced surface plasmon resonance (SPR) intensifies localized electromagnetic fields [22]. CM contributions originate from adsorbate-substrate interactions, including resonance Raman effects, charge-transfer, and interfacial chemical bonding, which synergistically augment molecular Raman signatures [23]. To improve SERS performance, researchers are dedicated to designing SERS-active substrates possessing superior stability, uniformity, reactivity, and high sensitivity. Benefiting from the synergistic development of nanoscience, optical technology, and electron transfer mechanism research, substrates with diverse shapes, compositions, and sizes can be synthesized. These substrates exhibit varying SERS enhancement factors (*Ef*s) and demonstrate significant potential in trace contaminant analysis [24]. Based on material composition and structure, SERS substrates are primarily categorized into monometal, bimetallic, and metal-nonmetal composite substrates. These substrates can significantly enhance Raman signals via both EM and CM mechanisms, thereby advancing SERS analysis.

### 2.1. Monometallic Substrates

**Figure 2:**
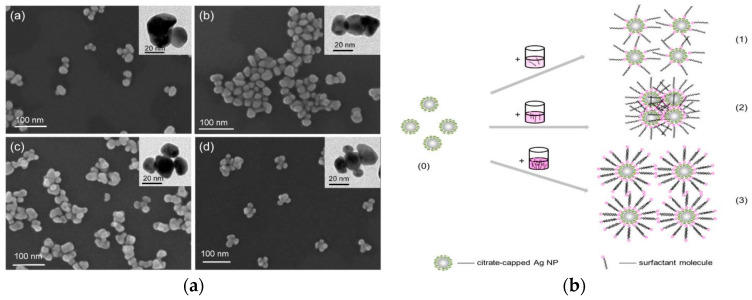
(**a**) Morphological characterization of Au nanoflowers [28]. Copyright Nanomaterials, 2021. (**b**) Cationic surfactants induce the aggregation of silver nanoparticles [32]. Copyright Microchemical Journal, 2021.

Monometallic nanomaterials exhibit unique physicochemical properties, attracting considerable research interest. Among various noble metal nanomaterials, Au and Ag nanomaterials are the most commonly employed SERS substrates, dominate as plasmonic SERS substrates due to their intense visible, near-infrared resonance and high enhancement factors [25]. Substrate performance is critically influenced by morphological parameters, crystallinity, and dimensions. Engineered nanostructures, including nanoparticles (NPs), nanospheres (NSs), nanoflowers (NFs), and nanorods (NRs), serve as highly active platforms for enhancing detection sensitivity toward food contaminants [26]. Au NPs are readily synthesized and exhibit good stability. More irregularly shaped nanomaterials generally exhibit stronger SPR, leading to more significant SERS signal enhancement [27]. Zhao et al. [28] synthesized colloidal Au NFs with controlled petal numbers via a template method (Figure 2a). The NF structures, featuring numerous corners, tips, and gaps, facilitate strong plasmonic coupling, generating abundant “hotspots” and markedly enhanced SERS intensity under 532 nm and 785 nm excitation wavelengths, achieving an *EF* 5.3 × 10^8^. The size of metallic nanoparticles is a paramount factor governing their SERS performance. It directly influences their localized surface plasmon resonance (LSPR) properties, which in turn dictates the electromagnetic enhancement efficiency [29]. As the size of gold or silver nanoparticles increases, their LSPR absorption peak red-shifts, enhancing SERS intensity. Sherpa et al. effectively tuned the size of Ag nanoparticles to a key range of 13–24 nm, resulting in controllable adjustments in LSPR wavelength (420–490 nm) and direct band gap (2.05–2.48 eV) [30]. These optimized optical properties correlate directly with the electromagnetic field enhancement strength, fundamentally enabling the *EF* to rise from 10^6^ to over 10^7^. Moreover, nanoparticle size is critical for hotspot formation. For the same gap distance, the SERS signal generated between larger nanoparticles (e.g., 60–80 nm) is significantly stronger than that between smaller nanoparticles (e.g., 20 nm). Luo et al. [31] fabricated a tunable Au/Au triangular nanogap array with gap sizes of 3–10 nm via atomic layer lithography. This array provides abundant “hotspots” (*EF*: 10^8^), exhibiting an SERS intensity significantly higher than that of a thin Au film of equivalent thickness. Furthermore, modifying substrate materials can also effectively enhance SERS “hotspots”.

Ag nanostructures are widely employed as SERS-active substrates due to their robust conductivity, oxidation resistance, biocompatibility, and cost advantage over Au, thereby significantly amplifying Raman signals. Hassan et al. [33] synthesized flower-like Ag NPs with highly roughened surfaces via a nucleation method, coupled with solid-phase extraction (SPE) for detecting methomyl, acetamiprid, and 2,4-D in green tea. The irregular surface morphology of the flower-like Ag NPs contains numerous crevices, which greatly enhance SERS intensity through localized plasmonic resonance. The incorporation of SPE allows rapid and efficient sample purification, accelerating the SERS detection process. The *EF* for AgNPs synthesized at 25 °C reached 1.39 × 10^6^. The detection limits (LODs) for methomyl, acetamiprid, and 2,4-D were as low as 1.88 × 10^−4^ µg/mL. Yao et al. [32] developed a surface-modified Ag nanoaggregates for SERS detection of hydrophobic contaminants in food (Figure 2b). The hydrophobicity of Ag nanoaggregates can be precisely tuned by controlling the alkyl chain length of cationic surfactants. This method offers well-defined parameters and excellent reproducibility, demonstrating promising application prospects.

### 2.2. Bimetallic Substrates

**Figure 3:**
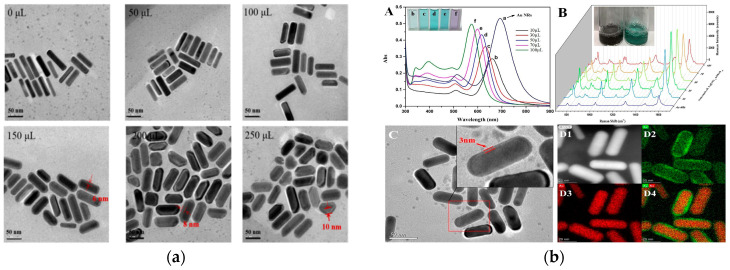
(**a**) Morphological characterization of AuNRs@Ag [36]. Copyright Sensors and Actuators B: Chemical, 2021. (**b**) Morphological characterization of Au@Ag NRs with the different addition volume of AgNO_3_ (A: UV–vis spectra and B: SERS spectra of Au@Ag NRs with the different addition volume of AgNO_3_ C and D1–D4: TEM spectra of Au@Ag NRs) [37]. Copyright Food Chemistry, 2022.

The combination of two plasmonic metals at ultrashort distances induces strong interfacial plasmonic coupling, thereby concentrating electromagnetic fields beyond monometallic limitations. These synergistic hotspots substantially amplify SERS signals and photocatalytic activity compared to single metal nanocrystals [34,35]. Au and Ag nanomaterials are the most common SERS substrates. Ag NPs exhibit sharper plasmonic peaks and provide higher Raman signals than Au NPs but are highly unstable. Combining Au and Ag into Au@Ag bimetallic nanostructures integrates the optical enhancement of Ag with the surface stability of Au, yielding excellent SERS performance. Yang et al. developed a reproducible method for synthesizing Au@Ag core–shell structures by systematically optimizing the volume of AgNO_3_. When 150 μL of AgNO_3_ solution was added, an optimal Ag shell with a thickness of approximately 6 nm was formed on the Au NR surface. Under these conditions, the resulting SERS substrate achieved the highest enhancement factor (2.91 × 10^7^) and exhibited excellent reproducibility across different batches (Figure 3a) [36]. Similarly, Chen et al. reported a reproducible approach for preparing core–shell Au@Ag NRs by controlling the amount of AgNO_3_ added [37]. The optimal plasmonic properties were achieved with the addition of 10 μL of AgNO_3_. The irregular surface of NRs provides higher Raman intensity than Au or Ag NSs (Figure 3b). Building on this, Xu et al. engineered magnetic Au@Ag NPs functionalized with 4-mercaptophenylboronic acid (4-MPBA), yielding Mau@Ag@MPBA substrate with enhanced SERS amplification (*EF*: 2.65 × 10^7^) [38]. NF structures, being more irregular than NRs, provide high-density “hotspots”, significantly boosting SERS substrate activity.

Moreover, SERS signal intensity depends critically on the homogeneity of “hotspots” and the distribution of molecular adsorption sites on the substrate [39]. To enhance detection specificity, Guo et al. designed a flexible SERS substrate incorporating Au@Ag core–shell nanocube arrays and MIP technology [40]. This platform demonstrates high sensitivity, outstanding selectivity, and mechanical flexibility, making it ideal for in situ detection on irregular surfaces. The Au@Ag nanocubes leverage the stability of the Au core and the strong plasmonic enhancement of the Ag shell, forming an ordered array with dense hotspots. This configuration ensures high signal sensitivity, uniformity, and stability. Coating the flexible substrate with MIP enables selective recognition and enrichment of target molecules from complex matrices, greatly improving selectivity and interference resistance. Consequently, the substrate performs reliably in real sample analyses with complex compositions. In summary, the SERS efficiency of noble metal nanostructures is highly influenced by their size, morphology, and architecture. The Ag shell contributes strong electromagnetic enhancement, while the Au core enhances chemical durability, preventing Ag oxidation and ensuring long-term stability and reproducible performance. For practical implementation, however, robustness in synthesis is as essential as high performance. Precise optimization of reaction parameters, such as precursor concentration and addition rate, enables the fabrication of SERS substrates with uniform morphology, consistent performance, and high reproducibility.

### 2.3. Metal-Nonmetal Composite Substrates

**Figure 4:**
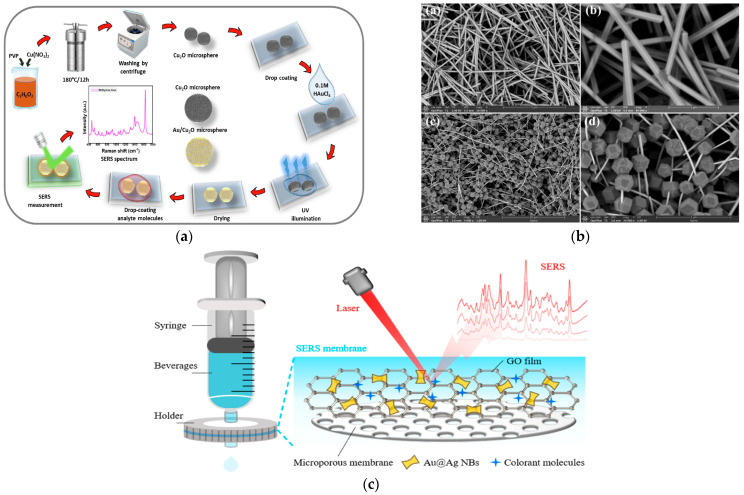
(**a**) Construction process of Au/Cu_2_O based SERS substrates for detection of RhB and MB [46]. Copyright Applied Surface Science, 2022. (**b**) Morphological characterization of AgNWs and AgNWs@ZIF-8 [49]. Copyright Food Chemistry, 2022. (**c**) GO/Au@Ag NBs-based SERS substrates for enrichment and detection of colorants in beverages [50]. Copyright Sensors and Actuators B: Chemical, 2021.

The recent development of novel non-metallic nanomaterials, including semiconductor materials, porous framework materials, and carbon-based nanomaterials in recent years, has opened new avenues for the engineering of SERS-active substrates [41,42,43]. Literature studies indicate that hybrid substrates combining metallic and non-metallic materials exhibit unique electronic and optical properties. Semiconductor materials, owing to their chemical stability, cost-effectiveness, and biocompatibility, making them promising candidates for SERS applications [44]. Constructing hybrid composites by integrating noble metal nanomaterials with semiconductors combines advantages such as high-density SERS hotspots and efficient mass transfer. Fueaimi et al. [45] fabricated silver nanofibers (Ag NFs) on Ag_2_S thin films using electron beam irradiation. The Ag_2_S semiconductor was selected for its unique properties and high stability. This design overcomes the susceptibility of silver to oxidation. Under optimal conditions, the Ag NF substrate demonstrated excellent SERS performance, achieving an *EF* as high as 1.57 × 10^6^ for methylene blue (MB) and a low LOD down to 1.18 × 10^−11^ M. Kumar et al. decorated Au NPs onto Cu_2_O microspheres using polyol and photoreduction methods to create a novel SERS substrate (Figure 4a) [46]. Examination of field emission scanning electron microscopy (FE-SEM) and elemental mapping images revealed rough copper oxide microspheres with an average particle size of 1.69 μm, uniformly decorated with Au NPs on the surface. The electromagnetic and chemical synergistic effects between the Au NPs, Cu_2_O microspheres, and target dye molecules significantly enhanced the SERS activity. After parameter optimization, *EF*s reached 2.55 × 10^12^ for rhodamine B (RhB) (LOD: 2.36 × 10^−13^ M) and 1.2 × 10^11^ for MB (LOD: 3.40 × 10^−12^ M). Wu et al. successfully synthesized ternary Au@CuO-Ag nanocubes with a core–shell-satellite structure via galvanic replacement [47]. This structure not only exhibited remarkable SERS enhancement but also demonstrated superior self-cleaning capability, maintaining high activity even after 6 cycles of use. Applied to malachite green detection, the LOD was as low as 10^−9^ M. Tiwari et al. developed an SERS substrate based on ZnO/ZnFe_2_O_4_ organic nanostructures modified with Au NPs [48]. X-ray photoelectron spectroscopy (XPS) was employed to characterize the surface chemical structure and bonding mechanisms of the synthesized ZnO/ZnFe_2_O_4_ nanocomposite. The results confirm that the electromagnetic interactions between Au^0^ and the material led to the enhancement of the SERS signal. The combination of ZnFe_2_O_4_, with its smaller band gap, and ZnO significantly enhanced the Raman signal (*EF*: 1.6 × 10^8^). This substrate achieved an LOD of 0.39 μM for melamine detection.

Although solid substrates like semiconductors provide compactness and uniformity for metallic materials, their practical application is often constrained by limitations including insufficient dispersibility, poor adsorption capacity, and suboptimal stability. Consequently, researchers have recently focused on developing novel porous flexible substrates to significantly enhance signal amplification and hotspot effects. Ding et al. [51] successfully fabricated an SERS-active substrate using a composite of MOFs and AgNPs (MOFs-AgNPs). This substrate enabled ultrasensitive detection of sildenafil (SIL) and pioglitazone hydrochloride (PIO) adulterants in dietary supplements. The enhanced performance stems from the strong inherent adsorption capability of MOFs, which effectively enriches target molecules, and the confinement of these molecules within the intense electromagnetic field enhancement zones of the in situ generated AgNPs. This combination significantly boosted SERS activity, resulting in MOFs-AgNPs substrates outperforming pure AgNP substrates. In another study, Yang et al. [49] engineered beaded AgNWs@ZIF-8 core–shell nanochains through controlled ZIF-8 growth on silver nanowires (Ag NWs), enabling in situ multiplex detection of methyl parathion and carbaryl residues on agricultural produce surfaces (Figure 4b). The XPS analysis revealed two distinct peaks for Ag 3d at Ag 3d_5/2_ and Ag 3d_3/2_, respectively. The spin–orbit splitting energy of 6.00 eV confirms the presence of metallic Ag (Ag^0^) in the AgNWs@ZIF-8 composite. This finding further indicates that the LSPR effect of metallic Ag generates a strong electromagnetic field, which amplifies the Raman signal of adsorbed molecules by a factor of 10^6^ to 10^10^. Benefiting from the microporous structure of the ZIF-8 shell and the SPR properties of the internal Ag NWs, the Ag NWs@ZIF-8 core–shell nanochain exhibited excellent adsorption capacity and significant SERS activity (*EF*: 4.2 × 10^7^). This synthesis method demonstrates excellent controllability and reproducibility, providing a new design concept for constructing novel metal NP@MOF composites. In summary, the porous structure of the MOF not only enriches the targets but also provides well-defined channels that serve as uniform anchoring sites for noble metal NPs. This effectively prevents NP aggregation and significantly enhances the substrate’s uniformity and signal reproducibility.

Carbon-based porous architectures, including porous carbon, graphene, and their derivatives, are fabricated through physical activation or chemical synthesis, offering enhanced surface activity. Relative to conventional non-porous materials, these architectures exhibit exceptional adsorption properties, enabling efficient enrichment of Raman “hotspots” and significantly enhancing detection sensitivity [52,53]. Graphene oxide (GO) and reduced graphene oxide (rGO) offer advantages such as stability, sensitivity, reproducibility, biocompatibility, ease of preparation, and modification. Furthermore, graphene demonstrates remarkable charge transfer between graphene and molecules [54,55]. However, given the limited contribution of chemical enhancement to SERS, graphene-metal hybrid architectures synergistically combine graphene-derived chemical enhancement with noble metal plasmonic amplification, generating significantly greater SERS intensity. Shanta et al. [56] developed a GO/Ag composite for detecting the hydrophobic pollutant PCB-77. This rGO-NP substrate outperformed planar silver nanoarrays and silver nanoprisms in SERS enhancement, achieving an LOD of 100 nM for coplanar PCB-77. Li et al. [57] developed a hydrophilic SERS imprinted sensor (AGP-MIM) by depositing MIPs onto an Ag/GO composite for antibiotic detection. SEM images showed that the synthesized composite exhibits a particle size of approximately 800 nm. The incorporation of GO further increases the surface roughness of the Ag/GO composite, which promotes the formation of a greater number of SERS “hotspots” [58]. The MIPs selectively adsorb target molecules on the surface while effectively protecting the internal SERS substrate. This platform achieved an LOD of 0.0078 nmol/L for antibiotics. He et al. [59] successfully fabricated magnetic Fe_3_O_4_/GO/Ag microspheres by coating Fe_3_O_4_ with GO followed by in situ Ag deposition, which was evidenced by SEM spectra. XPS spectrum confirm the presence of both Ag^0^ and the GO component. These exhibited higher SERS intensity than Fe_3_O_4_/Ag microspheres (LOD: 10^−9^ M) and possessed catalytic activity, completely degrading methylene blue and ciprofloxacin within 12 min. To achieve on-site detection of beverage colorants, Kong et al. [50] employed a GO/Au@Ag nanobox (NB)-functionalized filtration membrane in conjunction with a portable Raman spectrometer (Figure 4c). SEM observations indicate that the wrinkled two-dimensional membrane structure of GO offers abundant anchoring sites for the deposition of metal nanomaterials, leading to the formation of dense SERS hotspots. Compared with bare Au@Ag NBs, the GO/Au@Ag NRs membrane exhibited a 2.74-fold enhancement in SERS intensity. The irregular shape of Au@Ag NBs enhances SERS intensity, while GO exhibits strong enrichment capability for target analytes, achieving an LOD of 1.12 × 10^−9^ mol/L for R6G. In addition to improving sensitivity through the chemical enhancement mechanism, the incorporation of carbon-based materials such as GO leverages their high specific surface area and π-π interactions to selectively enrich hydrophobic target molecules. This effectively mitigates interference from hydrophilic compounds in complex food matrices, thereby improving the selectivity and robustness of the analysis.

Carbon dots (CDs), consisting of a sp^2^-hybridized carbon core and diverse surface functional groups, represent an emerging class of carbon-based nanomaterials [60,61]. Integration of CDs with metallic NPs leverages these structural attributes to achieve significant enhancement in SERS performance. Luo et al. [62] synthesized Au@CDs composites with an average diameter of 24 nm and a ~2 nm CD shell through the reduction of HauCl_4_ with CDs at 100 °C. The ultrathin CD layer promotes the adsorption of aromatic molecules, resulting in an *EF* of 8.8 × 10^3^, approximately 4 times higher than that of AuNPs. Applied to rhodamine 6G detection, the LOD was approximately 3.8 × 10^−7^ nmol/L. Bhunia et al. [63] fabricated a SERS sensor by depositing Ag NPs onto an active flexible polydimethylsiloxane (PDMS) film embedded with CDs. The incorporation of CDs markedly enhanced the SERS performance of the Ag NPs through efficient energy transfer between CDs and AgNPs. This platform achieved nanomolar-level detection of *Pseudomonas aeruginosa*. Chen et al. [64] prepared CDs-capped Ag NPs (AgNPs/CDs) with nanostructures including nanochains, nanoplates (2D), and nanobodies by controlling CD dosage. Transmission Electron Microscopy (TEM) and X-ray diffraction (XRD) analyses revealed distinct lattice fringes of the Ag NPs, while XPS and Fourier Transform Infrared Spectroscopy (FT-IR) spectra further confirmed the successful synthesis of both CDs and Ag NPs. The composites showed good dispersibility in water, and numerous tiny gaps (“hotspots”) formed between adjacent particles, greatly enhancing SERS activity. Among them, the 2D-AgNPs/CDs structure exhibited the highest SERS intensity, achieving an LOD of 3.9 ppt for thiram residues in apples. Additionally, modifying metal NPs with flexible substrates like cellulose or PDMS has also yielded satisfactory SERS intensity. Although CDs possess relatively weak plasmonic activity, their abundant surface polar functional groups facilitate the enrichment of target molecules through hydrogen bonding, electrostatic interactions, and π-π stacking, creating favorable conditions for charge transfer. Moreover, certain doped carbon dots can reduce the energy barrier associated with charge transfer, promoting more efficient electron exchange between the Ag substrate and target molecules, and leading to a significant enhancement of the Raman signal [65,66].

## 3. Applications of Noble Metal-Based Nanocomposites for SERS Detection of Food Contaminants

In food safety applications, noble metal-based nanomaterials have enabled novel SERS-based sensor platforms, facilitating rapid, sensitive, and non-destructive screening of diverse contaminants and additives (Table 1). These analytical tools detect trace pesticides, veterinary drugs, heavy metal ions, biotoxins, and food additives, which is critical capabilities for ensuring food safety. They enable the identification and quantitative analysis of harmful substances prior to their entry into the consumption stage [67,68].

### 3.1. Biotoxins

Biotoxins are a class of naturally occurring toxic substances derived from living organisms, such as animals, plants, or microorganisms, capable of exerting specific toxic effects in other biological species. These toxins readily contaminate human food chain, causing acute or chronic poisoning. Moreover, their potential long-term health risks, including mutagenicity and teratogenicity, pose serious threats to human health [85,86]. SERS technology, leveraging its ultra-high sensitivity, excellent specificity, and capability for multi-target synchronous detection, offers significant advantages in the field of biotoxin analysis. Advances in nanotechnology have further facilitated functional nanomaterial design. These nanomaterials can amplify SERS signals, enhance detection sensitivity, and expand applicability to complex real samples [87,88]. Owing to these attributes, SERS has emerged as a promising tool for detecting biotoxins such as mycotoxins, microcystins, and tetrodotoxin. Current research is increasingly focused on developing novel nanomaterials and methods to achieve higher sensitivity and more rapid detection performance.

**Figure 5:**
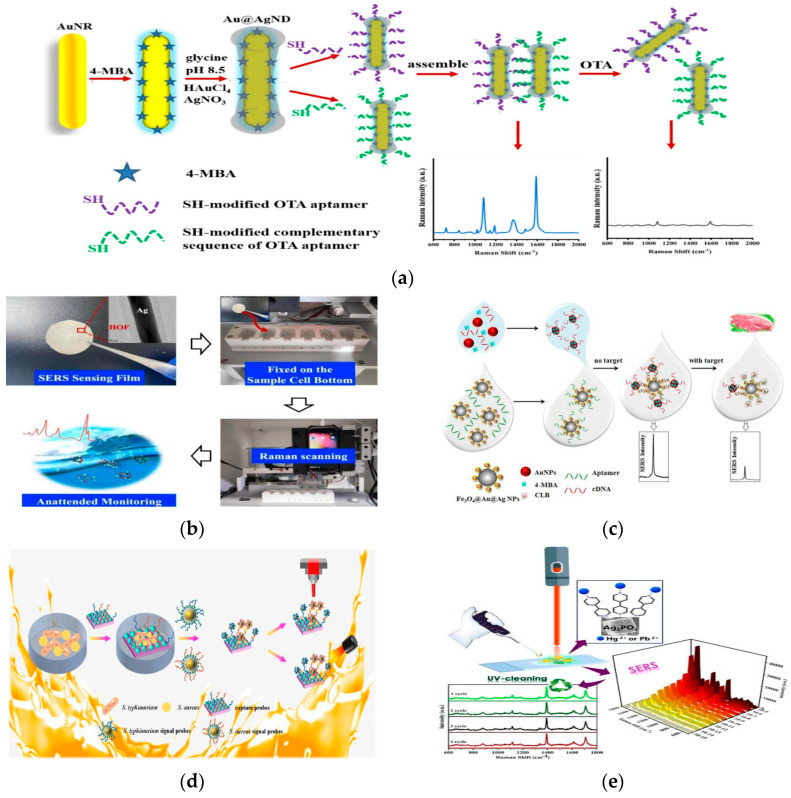
(**a**) Au@Ag nanodumbbell based inter-nanogap SERS aptasensor for the detection of OTA [71]. Copyright Analytica Chimica Acta, 2021. (**b**) Based on AgMW@HOF SERS substrates for detecting BPE [76]. Copyright Chemical Engineering Journal, 2024. (**c**) SERS-based aptasensor for the detection of clenbuterol hydrochloride [78]. Copyright LWT, 2020. (**d**) ZnO/Ag-Au@Ag SERS probes to detect *S. typ* and *S. aureus* [81]. Copyright Analytica Chimica Acta, 2025. (**e**) Ag_3_PO_4_/SWCNT as efficient SERS substrate for detecting Hg^2+^ and Pb^2+^ [82]. Copyright Journal of Colloid and Interface Science, 2022.

Currently, SERS-based toxin detection platforms have been widely established, with aptamer-mediated specific recognition emerging as the predominant strategy due to its superior selectivity. Ma et al. designed a dumbbell-shaped Au@Ag nanodumbbell (Au@Ag ND) aptasensor for ultrasensitive detection of ochratoxin A (OTA) (Figure 5a) [71]. TEM showed that glycine induced preferential growth of Ag on the ends of Au NRs, forming dumbbell-shaped Au@AgNDs with the thickness of the outer Ag shell at 5 nm. Further characterization by Ultraviolet-Visible Near-Infrared spectroscopy revealed an absorption peak around 400 nm, indicating the successful deposition of the silver shell onto the gold nanorod surface, which is consistent with the TEM observations. Subsequently, SH-Apt and SH-cDNA were covalently modified onto opposite ends of the Au@AgNDs. Base pairing formed nanogap structures generating high-density hotspots. Upon OTA binding, the aptamer dissociated from the complementary strand, causing the disintegration of the dumbbell structure and consequent SERS signal reduction. The LOD of 0.007 ng/mL is lower than those reported for conventional HPLCs (typically 0.03 μg/mL) [72]. Although SERS technology demonstrates exceptional performance for detecting standard solutions in laboratory settings, its application in real complex food matrices, such as meat, fruits, and beverages, remains challenging. Complex food components can cause strong background fluorescence, spectral interference, and nonspecific adsorption, which mask the SERS signals of target molecules and compromise detection accuracy and reproducibility [89]. In comparison, magnetic composite materials allow for rapid magnetic separation and concentration, substantially streamlining sample preprocessing. This integrated approach markedly improves the anti-interference performance, sensitivity, and operational efficiency of SERS technology in real industrial applications. He et al. [90] designed an efficient magnetic separation SERS platform utilizing SH-cDNA-modified Fe_3_O_4_@Au NFs as capture probes and SH-Apt/Cy3-Apt-modified Au@Ag NSs as reporter probes. Surface characterization techniques such as SEM, zeta potential, and XRD confirmed the successful fabrication of both the SERS substrate and the probes. Strong SERS signals were generated via base pairing between the aflatoxin B_1_ (AFB_1_) aptamer and its complementary strand. In the presence of AFB_1_, the aptamer preferentially binds the toxin, leading to weakened signals after magnetic separation. The method achieved an LOD of 0.40 pg/mL and a recovery rate of 96.6% to 115%.

To overcome the limitations of single-aptamer sensors, such as susceptibility to background interference and poor reliability, Wang et al. developed a photothermal (PT) and SERS dual-mode immunochromatographic sensor for sensitive AFB_1_ detection [91]. This study constructed bayberry-like core-satellite nanostructures loaded with DTNB and conjugated them with antibodies to form a dual-functional photothermal-SERS probe (PT@SERS NPs), enabling both qualitative and quantitative detection of AFB_1_. The resulting probe demonstrated a photothermal conversion efficiency of 42.11% and high SERS intensity (*EF*: 1.59 × 10^7^). The LOD of this assay was 0.0073 ng/mL, which was lower than those reported for conventional HPLC (0.07 ng/mL) and colorimetric (0.008 ng/mL) assay [92], but was higher than apt-based strategy (0.40 pg/mL) [90].The constructed SERS sensor demonstrated exceptional dual performance, offering a promising technological approach for rapid mycotoxin detection.

### 3.2. Pesticides and Veterinary Residues

Pesticides are widely applied in agriculture for pest control, weed management, and crop growth regulation, while veterinary drugs are employed for preventing and treating animal diseases and promoting growth. However, residues of these agrochemicals may persist in food and accumulate in human body, posing potential health risks. Therefore, developing rapid, simple, and highly sensitive detection technologies for pesticide and veterinary drug residues is imperative [93,94]. In the field of pesticide detection, SERS technologies offers significant advantages over traditional chromatographic methods. The latter often requires complex sample preparation, being time-consuming, labor-intensive, and costly. In contrast, SERS enables rapid, non-destructive, and in situ analysis, making it well-suited for on-site screening. Numerous innovative SERS strategies have been developed for trace detection of pesticide and veterinary drug residues. Yang et al. [36] constructed a magnetic separation-assisted SERS immunosensing platform using core–shell AuNRs@Ag. Leveraging the property that AuNRs facilitate “hotspot” formation more readily than spherical NPs for Raman signal enhancement, 4-mercaptobenzoic acid (4-MBA) as internal standard signal molecule were stably sandwiched between the Au core and Ag shell. The optimal AgNO_3_ addition was determined by monitoring the variation in silver shell thickness observed in TEM images. When 150 μL of AgNO_3_ solution was added, an optimal Ag shell thickness of approximately 6 nm was achieved on the surface of the AuNRs, resulting in a maximum Raman *EF* of 2.91 × 10^7^. This method employed amantadine (AMD)-BSA modified AuNRs@Ag as signal probes combined with monoclonal antibody-conjugated magnetic beads (MBs) as capture probes. Sensitive detection of the veterinary drug AMD was realized via immunocompetitive reaction (LOD: 0.0038 μg/L). Magnetic separation significantly improved detection efficiency, enabling completion of the entire process within 30 min.

However, antigen–antibody binding can lead to false-positive results. Aptamers, owing to their high specificity and precise recognition capabilities, have emerged as more reliable molecular recognition tools. He et al. developed a multifunctional nanoprobe (CDNAg@MIPApt) for ultrasensitive glyphosate detection [73]. A glyphosate-imprinted MIP provides cavities with complementary size and functional groups, ensuring specific target capture. In this work, the successful recognition by the MIP is the key trigger for the detection signal change. Glyphosate binding inhibits the probe’s catalytic activity, reducing AgNP generation and decreasing all three optical signals. This “signal-off” response shows a linear negative correlation with concentration, achieving sensitive detection of glyphosate with an LOD of 0.034 nmol/L, which is lower than those reported for conventional HPLC (1.76 μmol/L) [95] and fluorescent (0.048 μmol/L) [81] techniques.

Porous framework materials, characterized by ultrahigh porosity, enormous specific surface area, and tunable pore size, can be grown in situ on the surface of metal NPs to form core–shell structures, significantly enhancing the sensing performance and stability of SERS substrates. Pu et al. constructed a 3D carbon cloth/ZnO-Ag@ZIF-8 SERS substrate [96]. The ZIF-8 coating offers high surface area and abundant adsorption sites, enhancing analyte enrichment. Within the 3D structure, ZIF-8 synergizes with ZnO and AgNPs to improve electromagnetic field distribution, increasing SERS sensitivity by two orders of magnitude compared to uncoated substrates. Sun et al. successfully synthesized a novel core–shell material (Ag MW@HOF) under mild conditions for SERS detection of the pesticide 1,2-bis(4-pyridyl)ethylene (BPE) residue (Figure 5b) [76]. The material composed of a silver microwire (Ag MW) core and a hydrogen-bonded organic framework (HOF) shell. This study revealed that the SERS sensing performance was significantly enhanced through the synergistic effects of surface hydrogen bonding, adsorption energy differences, and notably, the pore confinement effect within the HOF shell. By precisely controlling the HOF shell pore size, the AgMW@HOF substrate exhibited highly active and quantifiable SERS responses to probe molecules within a concentration range of 200–1000 ng/mL. Additionally, a HOF-based core–shell SERS chip was fabricated for on-site monitoring of pesticides in aqueous environments. Owing to the mild synthesis conditions, excellent stability and tunable structure, porous framework materials represent promising and controllable platforms for constructing functional SERS substrates. Importantly, the clarification of the SERS signal enhancement mechanisms provides valuable insights for the further development of HOF-based SERS sensing technologies.

### 3.3. Illegal Food Additives

Illegal food additives refer to non-edible chemical substances (e.g., melamine, clenbuterol, industrial dyes) incorporated during food processing. These substances are prohibited due to their significant toxicity or unknown hazards [97]. By utilizing plasmonic enhancement in Au/Ag/Cu nanomaterials, SERS intensifies molecular Raman signatures, enabling ultra-sensitive detection of illegal additives. Melamine, an industrial chemical, is often fraudulently incorporated into dairy and feed products to artificially elevate apparent protein content owing to its high nitrogen content (66%) [98]. While exhibiting low acute toxicity, excessive intake leads to the formation of insoluble melamine-cyanuric acid crystals in the kidneys, inducing renal failure. Xing et al. [77] developed an aptamer-modified substrate with in situ synthesized Ag NPs for simultaneous detection of melamine and cyromazine in raw milk. The aptamer functioned as a recognition element, enabling specific binding and precise capture of target molecules. The achieved LODs were 43.5 ppb for melamine and 23.6 ppb for cyromazine, both well below the relevant national standard limits. The entire detection process can be completed within 10 min, facilitating real-time and on-site analysis. Dong et al. [99] constructed a highly sensitive melamine detection platform using aptamer-conjugated SERS nanosensors combined with oligonucleotide microarray technology, designed for trace analysis in milk. The sensor utilized Au NPs functionalized with Raman reporters and poly-thymine aptamers, enabling specific melamine recognition via the formation of a “T-M-T” hydrogen-bonding structure. The LOD of this assay was 1.0 ppt, which was even lower than those reported for conventional HPLC (0.73 ppm) [100] and Ag NPs-Apt (43.5 ppb) assay [77].

Clenbuterol was originally developed for asthma treatment but has been illegally used as a feed additive to promote leanness in livestock. Due to its slow metabolism and persistence in animal tissues, clenbuterol has caused severe food poisoning incidents. As a result, the use of clenbuterol in livestock production has been banned in China and the European Union (EU) [101,102]. Recently, various SERS sensing strategies have been established for detecting trace clenbuterol. Zhu et al. [103] developed a competitive immunoassay by conjugating antibodies to 4,4′-dipyridyl (4,4′-DP)-labeled Au NPs and immobilizing clenbuterol-BSA on a glass substrate. Clenbuterol in samples competed with the immobilized clenbuterol-BSA for binding to the antibody-conjugated NPs. The proposed SERS method enabled accurate detection of clenbuterol in swine urine with a LOD of 0.1 pg/mL. To address possible false positives associated with antigen–antibody binding and to improve sensitivity, Duan et al. [78] modified aptamers onto Fe_3_O_4_@Au@Ag magnetic NPs as capture probes, and introduced 4-MBA-labeled Au NPs bound to complementary DNA as signal probes (Figure 5c). The specific hybridization between aptamer and complementary DNA generated a strong SERS signal. Upon introduction of clenbuterol, the target analyte bound to the aptamer, competitively displacing the signal probe and resulting in signal reduction. The method achieved an LOD of 0.003 ng/mL for clenbuterol detection in pork samples. Cheng et al. [104] innovatively employed a rGO/AuNPs composite as the SERS probe. TEM observations confirmed that surface chemical modification of GO significantly improved the electrostatic adsorption and nucleation of AuNPs. The rGO not only enhanced the SERS effect but also enriched target molecules via π-π interactions and improved substrate stability. Utilizing liquid–liquid extraction for urine sample pretreatment, this method exhibited an LOD of 0.5 ng/mL and a total analysis time of 8 min, rendering it amenable to point-of-care testing.

Malachite green (MG) is a triphenylmethane dye once widely used in aquaculture to inhibit microbes, fungi, and parasites. However, its use is now prohibited due to its carcinogenic, teratogenic, and mutagenic risks to humans. For detecting trace MG residues in aquatic products, Liu et al. [79] utilized sea urchin-like SG@SiO_2_ NPs as a SERS substrate. The numerous tips on this nanostructure generated significant SERS signals via localized surface plasmon resonance (*EF*: 3.2 × 10^6^). However, pure SG NPs exhibited poor stability, depositing a silica layer effectively enhanced substrate stability. Successfully applied to MG detection in tilapia, the LOD reached 1.5 × 10^−9^ mol/L, which was lower than those reported for immunoassay (1.29 × 10^−7^ mol/L) [105] and conventional HPLC-MS/MS (5.48 × 10^−9^ mol/L) technology [106]. To further improve selectivity and enrichment efficiency, Ekmen et al. [107] employed MIP technology, which offers abundant specific recognition sites and strong binding affinity. Magnetic NPs coated with an MIP layer were prepared for specific capture and enrichment of MG. Subsequently, the magnetic complex was then deposited onto a silicon wafer coated with Ag nanodendrites for SERS detection. This enabled highly sensitive analysis of MG in carp, achieving an LOD of 1.62 × 10^−6^ M.

### 3.4. Foodborne Pathogens

Foodborne pathogens are bacteria capable of causing food poisoning or using food as a transmission route. They can enter the human body through contaminated water or food, subsequently causing foodborne illnesses. These pathogens have become one of the most critical global public health issues. Common foodborne pathogens include *Escherichia coli* (*E. coli*), *Salmonella Typhimurium* (*S. Typhimurium*), *Staphylococcus aureus* (*S. aureus*), and *Listeria monocytogenes* (*L. monocytogenes*) [108,109]. Furthermore, Chuesiang et al. leveraged the coffee-ring effect for SERS signal enhancement, depositing bacteria, aptamer, AgNPs onto gold-coated slides to form coffee rings prior to SERS detection [69]. This enabled detection of *Salmonella Enteritidis* in ground beef at levels as low as 4 × 10^4^ CFU/g within 4 h. Building on this, Zhao et al. developed a multifunctional Fe_3_O_4_@Au-Apt sensor integrating capture, detection, and photothermal therapy [110]. The high surface roughness of Fe_3_O_4_@Au improved SERS signals, while Fe_3_O_4_ magnetic separation accelerated the reaction process. The aptamer-conjugated Fe_3_O_4_@Au-Apt specifically captured target bacteria, achieving an LOD of 25 CFU/mL for *S. aureus*, which is lower than that of polymerase chain reaction (5 × 10^3^ CFU/g) [111]. Additionally, this substrate exhibited strong photothermal conversion efficiency; temperature increased by 30.8 °C within 5 min under laser irradiation, killing up to 97% of the target bacteria.

To further improve multiplex detection capability, Cheng et al. constructed a magnetic-assisted platform utilizing wheat germ agglutinin (WGA)-modified magnetic NPs for efficient bacterial capture [74]. With streptavidin (SA) mediation, this platform significantly increased the number of aptamer-conjugated AuNPs, enabling simultaneous and highly sensitive detection of *S. aureus* and *L. monocytogenes* as limits as low as 3 and 5 cells/mL, respectively. Addressing the issue of weak binding between bacteria and metal NPs, Wang et al. [75] designed a boronic acid-functionalized, polydopamine-coated Au@Ag nanoprobe for detecting *S. aureus* and *E. coli*. The boronic acid groups effectively captured bacteria, whereas the application of a biocompatible polydopamine (PDA) coating stabilized SERS signals and protected the MNPs from oxidation. Combined with IgG@Fe_3_O_4_ magnetic separation, this probe amplified the SERS signal by 10^8^-fold upon bacterial binding, successfully classifying multiple pathogens with a LOD of 10 CFU/mL. And the entire detection process was completed within 30 min. Zhou et al. [80] proposed an integrated bacterial SERS detection platform combining magnetic capture, free antibodies, and PA-SERS tags. In this method, target bacteria were first captured by Fe_3_O_4_@Au-Apt magnetic beads, then labeled with free antibodies that provided Fc sites, and finally bound to PA-SERS tags. This strategy circumvented antibody inactivation associated with direct tag conjugation and reduced steric hindrance, significantly improving the specificity and sensitivity for detecting *E. coli*, *L. monocytogenes*, and *S. Typhimurium* (LODs: 10, 10, and 25 CFU/mL, respectively), representing a hundred-fold improvement over traditional lateral flow immunoassay (LFA). However, most current studies focus solely on constructing single-function biological platforms for accurate analysis of foodborne pathogens. Dai et al. developed an integrated SERS platform based on a “capture probe/bacteria/signal probe” sandwich structure (Figure 5d) [81] SEM images revealed that the roughness of the SERS substrate significantly increased due to the deposition of Ag nanocrystals, which indirectly confirms the successful preparation of ZnO/Ag nanocomposites. Utilizing aptamer-modified ZnO/Ag and Au@Ag-4-MPBA-aptamer as probes, this sensor successfully achieved simultaneous, highly sensitive detection of the foodborne pathogens *S. Typhimurium* and *S. aureus* (LOD: 10 CFU/mL) and efficient photothermal sterilization (photothermal efficiency of 54.32%). This sensor integrates dual electromagnetic and chemical enhancement mechanisms, significantly boosting SERS signal intensity (*EF*: 4.67 × 10^5^). Its inherent biocompatibility renders it a novel tool for food safety detection and mitigation applications.

### 3.5. Heavy Metal Ions

Heavy metal ions, such as Hg^2+^, Pb^2+^, Cd^2+^, Cr^3+^, and Cu^2+^, pose serious threats to human health and ecological environments due to their high toxicity, persistence, and bioaccumulative nature [112,113]. SERS-based assays are now widely used for trace-level monitoring of these contaminants, capitalizing on exceptional sensitivity, rapid response kinetics, and molecular fingerprinting capabilities. The integration of noble metal-based nanomaterials as SERS substrates, which possess unique optical properties and high specific surface area, further improves analytical performance to nanogram-level detection limits. These advances open new avenues and establish a highly sensitive framework for contaminant monitoring and bioanalysis.

For instance, Kamal et al. developed a silver phosphate (Ag_3_PO_4_) microcube SERS substrate using a double precipitation method for the ultrasensitive detection of Hg^2+^ and Pb^2+^ [82]. By synergistically immobilizing target ions with the organic linker 4,4′-bipyridine (BPy), this substrate achieved exceptional sensitivity (*EF*: 10^10^) and an ultra-low detection limit (10^−15^ M). Beyond exhibiting excellent stability and selectivity, the Ag_3_PO_4_ substrate also demonstrated outstanding catalytic degradation capability, allowing for at least 4 cycles of reuse under light illumination. These properties render it an efficient and reliable platform for recyclable SERS detection of heavy metal ions (Figure 5e). To increase the density of plasmonic nanostructures and interaction sites, three-dimensional (3D) cross-linked structures have been developed, which offer higher electromagnetic field enhancement than traditional two-dimensional (2D) substrates. Following this approach, Parveen et al. developed a novel 3D SERS substrate, ZnO@single-walled carbon nanotubes (ZnO@SWCNTs), for environmental heavy metal ion detection [83]. Surface morphology techniques such as FE-SEM, and XRD demonstrated that the necklace-like and pearl-like structures of ZnO NPs were successfully formed on the surface of SWCNTs. The 3D architecture greatly increases the coverage of plasmonic nanostructures and the density of hot spots, leading to a strong SERS effect. Furthermore, SWCNTs offer a much larger surface area than 2D planar structures, allowing for the adsorption of more heavy metal ions and further improving detection sensitivity. This substrate showed high sensitivity and selectivity for Pb^2+^ with an ultra-low LOD of 0.225 nM, excellent stability, and good reproducibility, making it a powerful tool for trace Pb^2+^ detection in aqueous solutions. Furthermore, He et al. [84] developed a novel plasmonic MOF-based SERS sensor (Ag@UiO-68-SMe) using an electrochemical method, which enabled the large-scale fabrication of nanotextured Ag needles. SEM observations confirmed the uniform growth of the nanotextured Ag needles on the MOF surface. The thiomethyl groups in the functionalized MOF specifically recognize Hg^2+^, capturing and concentrating it from complex samples. This greatly enhances the local Hg^2+^ concentration at the sensing interface, achieving highly sensitive on-site detection with a LOD of 0.17 ppb, representing a three-fold improvement over conventional HPLC-inductively coupled plasma-MS (HPLC-ICP-MS) [114].

However, Raman signal amplification strategies that rely on complex nanostructures can introduce substantial measurement variability. This affects the reproducibility of SERS measurements and hinders the establishment of standardized detection protocols. Moreover, such strategies often exhibit good performance only for specific target molecules, further limiting the technique’s general applicability. While machine learning (ML) algorithms offer potential solutions to these challenges, the absence of standardized model development pipelines and benchmark datasets remains a significant obstacle. To address this, Park et al. constructed a benchmark SERS spectral dataset using lead nitrate (Pb(NO_3_)_2_) and conducted a comparative study to identify the optimal combination of preprocessing steps and ML models [115]. By evaluating the classification performance of multiple ML models, their optimized model successfully identified Pb^2+^ in independent testing and achieved a balanced accuracy of 84.6% in cross-batch validation.

## 4. Challenges and Future Perspectives

Noble metal-based nanomaterials demonstrate considerable potential for highly sensitive detection of food contaminants by leveraging their unique SPR effect to significantly enhance Raman scattering signals. However, their practical application is accompanied by both promising prospects and significant challenges.

(1) Material stability and detection reproducibility: The stability of noble metal NPs, which directly influences the reproducibility and reliability of SERS detection strategies, is affected by the preparation techniques and storage conditions. Due to their high surface energy and susceptibility to oxidation, these NPs are prone to aggregation in complex matrices or during storage, leading to weakened SPR effects and diminished SERS signals. Consequently, developing reproducible and robust chemical production schemes is essential to address this challenge. Noble metal composites offer a key advantage over single materials. They provide a multifunctional platform that improves SERS reproducibility, interference resistance, and robustness. Moreover, immobilizing NPs onto porous supports via covalent bonding or coordination interactions can effectively prevent aggregation. Precise control over nanoparticle size monodispersity is crucial for the fabrication of reproducible and reliable SERS substrates. Therefore, when designing SERS substrates for food contaminants, the size of the metallic nanoparticles must be carefully tailored to achieve optimal analytical performance.
(2) Specificity in complex systems: The co-existence of multiple contaminants in food, including structurally similar compounds, makes accurate discrimination difficult when relying solely on non-specific adsorption onto noble metal nanomaterials. To address this limitation, the integration of recognition elements (e.g., antibodies, aptamers, MIPs) or microfluidic devices can significantly improve the sensitivity and selectivity of quantitative detection for target molecules. Additionally, improving signal resolution by combining SERS with advanced techniques, such as confocal micro-Raman or surface-enhanced resonance Raman spectroscopy followed by data analysis using partial least squares-discriminant analysis (PLS-DA) or deep learning algorithms to extract characteristic features from overlapping peaks, represents a promising strategy.
(3) Mitigation of matrix interference: The complexity of food matrices can cause severe interference in SERS detection, potentially masking target signals and impeding accurate identification and quantification. External sample pretreatment, such as dilution, centrifugation, organic solvent extraction, or SPE, can also be utilized to purify and concentrate target analytes. On the other hand, a viable solution involves the development of targeted purification materials with high adsorption capacity. These materials can selectively capture target analytes through surface-specific interactions, such as molecular imprinting or coordination binding within porous framework structures, thereby minimizing the matrix contact area and interference.
(4) Scalable and cost-effective fabrication: There is an urgent need to develop scalable and cost-effective nanomaterial manufacturing techniques. The key lies in the selection of straightforward and environmentally benign synthesis approaches and the exploration of integration strategies compatible with existing commercial technologies, both of which offer substantial potential for reducing production costs. Flexible SERS substrates, due to their portability and versatility, are particularly well-suited for large-scale practical applications.

## Figures and Tables

**Table 1:** A summary of various noble metal-based nanocomposites as substrates for SERS detection of food contaminants.

SERS Substrates		Contaminants	*EF*s	Detection Performances	Ref.
Monometal	Au NRs	Thiabendazole	-	Linear range: 1–18 μM LOD: 0.33 μg /mL Recovery: 83.5–98.5% (Citrus)	[27]
	Au NFs	Methyl Parathion	1.54 × 10^8^	LOD: 31.56 ng/cm^2^	[29]
	Ag NPs	*S. Enteritidis*	-	Linear range: 3–6 log CFU/mL LOD: 4 log CFU/mL	[69]
	Au NPs@4-MBA	Cd^2+^/Cu^2+^/Ni^2+^	2.1 × 10	LOD: <1 μM	[70]
Bimetal	AuNRs@Ag	Amantadine	2.91 × 10^7^	Linear range: 0.01–50.0 μg/L LOD: 0.0038 μg/L Recovery: 82.0–106.0% (Chicken/Egg/Milk)	[36]
	Au@AgND	OTA	1.7 × 10^5^	Linear range: 0.01–50 ng/mL LOD: 0.007 ng/mL Recovery: 92.4–101.6% (Peanut oil)	[71]
	Au@Ag NPs	Kanamycin	-	Linear range: 10–100 ng/mL LOD: 0.90 pg/mL Recovery: 90.4–112% (Liquid whole milk)	[72]
	HAu@AgNFs@MBA	2,4-dichlorophenoxyacetic acid	3.28 × 10^8^	Linear range: 0.001−100 μg/mL LOD: 0.11 ng/mL Recovery: 89.73–100.27 % (Tea/Milk)	[73]
	Au@Ag@PDA	*S. aureus*/*E. coli*/*S. dysenteriae*/ *P. aeruginosa*/*K. pneumonia*	2.92 × 10^8^	Linear range: 10^3^–10 CFU/mL LOD: 10 CFU/mL	[74]
	Au/Ag nanodimers	*S. Typhi*/*S. aureus*	-	Linear range: 10^2^–10^7^ CFU/mL LOD: 50 CFU/mL (*S. typhimurium*); 96 CFU/mL (*S. aureus*) Recovery: 92.86–107.32% (Milk)	[75]
Metal-nonmetal	AgNWs@ZIF-8	Methyl parathion/ Carbaryl	4.2 × 10^7^	LOD: 7.6 × 10^−9^ mol/L (Methyl parathion); 5.7 × 10^−9^ mol/L (Carbaryl) Recovery: 77.4–117.5% (Apple/Cabbage/Strawberry)	[49]
	Au@HgNPs/CDs	Hg^2+^/AFB_1_	-	Linear range: 0.625–90 µg/L LOD: 0.147 µg/L (Hg^2+^); 0.08 µg/L (AFB_1_) Recovery: 89.15–109.63% (Peanut oil)	[61]
	AgMW@HOF	1,2-bis(4-pyridyl) ethylene	-	Linear range: 200–1000 ng/mL	[76]
	CYR-AgNPs	Melamine/Cyromazine	-	Linear range: 0.1–0.5 ppm LOD: 43.5 ppb (Melamine); 23.6 ppb (Cyromazine) Recovery: 95–105% (Raw milk)	[77]
	Fe_3_O_4_@Au@Ag	Clenbuterol hydrochloride	-	Linear range: 0–1.5 ng/mL LOD: 0.003 ng/mL Recovery: 90.7–108.0% (Pork)	[78]
	Au@SiO_2_	Malachite green	3.2 × 10^6^	Linear range: 10^−5^–10^−9^ M LOD: 1.5 × 10^−9^ M Recovery: 91.69–102.49% (Tilapia filets)	[79]
	Fe_3_O_4_@Au	*E. coli*/*L. mono*/*S. typhi*	-	Linear range: 10^7^–10 cells/mL LOD: 10 cells/mL (*E. coli*); 10 cells/mL (*L. mono*); 25 cells/mL (*S. typhi*) Recovery: 84.0–110.2% (Milk/Lettuce/Urine)	[80]
	ZnO/Ag–Au@Ag	*S. Typhi*/*S. aureus*	4.67 × 10^5^	Linear range: 10–10^8^ CFU/mL LOD: 10 CFU/mL	[81]
	Ag_3_PO_4_/SWCNT	Hg^2+^/Pb^2+^	10^10^	Linear range: 10^−5^ M–10^−15^ M LOD: 10^−15^ M Recovery: 97.66–98.85% (Water/River)	[82]
	ZnO@SWCNTs	Pb^2+^	-	Linear range: 0.01–100 μM LOD: 0.225 nM	[83]
	Ag@UiO-68-SMe	Hg^2+^	-	LOD: 0.17 ppb	[84]

Note: “-” in the table indicates that the relevant content is not explicitly mentioned in the document.

## Data Availability

No new data were created or analyzed in this study.
